# Comparative Analysis of Single‐ and Dual‐Marker Strategies for Rapid Non–ST‐Segment–Elevation Myocardial Infarction Rule‐Out Using Cardiac Myosin‐Binding Protein C, Copeptin, and High‐Sensitivity Cardiac Troponin T in the Emergency Department

**DOI:** 10.1161/JAHA.124.039379

**Published:** 2025-05-13

**Authors:** Mustafa Yildirim, Christian Salbach, Matthias Mueller‐Hennessen, Norbert Frey, Evangelos Giannitsis

**Affiliations:** ^1^ Department of Internal Medicine III, Cardiology University Hospital of Heidelberg Heidelberg Germany; ^2^ DZHK (German Centre for Cardiovascular Research), Partner Site Heidelberg/Mannheim Heidelberg Germany

**Keywords:** acute coronary syndrome, cardiac myosin‐binding protein C, cardiac troponin, emergency department, high‐sensitivity, Biomarkers, Myocardial Infarction

## Abstract

**Background:**

This study compared the diagnostic and prognostic performance of various non–ST‐segment myocardial infarction (NSTEMI) rule‐out protocols, incorporating cardiac myosin‐binding protein C (cMyBP‐C), high‐sensitivity cardiac troponin T (hs‐cTnT), and Copeptin, both individually and as part of dual‐marker strategies (DMSs) against the European Society of Cardiology 0/1‐hour and 0/3‐hour algorithms.

**Methods:**

We enrolled 1765 patients presenting to the emergency department with suspected NSTEMI. We evaluated biomarker algorithms including cMyBP‐C (<10 ng/L, <2.3 ng/L), hs‐cTnT (limit of blank [<3 ng/L], limit of detection [<5 ng/L], 99th percentile [≤14 ng/L]), and DMS combinations of copeptin (<10 pmol/L) with hs‐cTnT, cMyBP‐C with hs‐cTnT, and copeptin with cMyBP‐C. The European Society of Cardiology 0/1‐hour and 0/3‐hour algorithms were also tested. We calculated negative predictive values and sensitivities for NSTEMI rule‐out and assessed effectiveness and prognostic performance based on cardiovascular events within 30 days and 1 year.

**Results:**

The areas under the curve were 0.922 for hs‐cTnT, 0.917 for cMyBP‐C, and 0.624 for copeptin in diagnosing NSTEMI. DMS protocols showed negative predictive values of 99.1% to 100%, comparable with the European Society of Cardiology algorithms (99.3%–100%). Sensitivities for DMS ranged from 96.2% to 100%. All protocols had low rates of the combined end point of cardiovascular events within 30 days (0.0%–0.6%).

**Conclusions:**

The European Society of Cardiology 0/1‐hour algorithm and DMS combining hs‐cTnT with either cMyBP‐C or copeptin provide highly reliable and safe protocols for NSTEMI rule‐out. These DMS approaches offer promising alternatives to current standards, potentially improving clinical decision making and efficiency in emergency departments.

**Registration:**

URL: https://clinicaltrials.gov; Unique identifier: NCT06128317

**Nonstandard Abbreviations and Acronyms**
cMyBP‐C: cardiac myosin‐binding protein C
DMS: dual‐marker strategy
ESC: European Society of Cardiology
FNR: false‐negative rate
GRACE: Global Registry of Acute Coronary Events
HighSTEACS: High‐Sensitivity Troponin in the Evaluation of Patients With Suspected Acute Coronary Syndrome
LoB: limit of blank
LoD: limit of detection
MIR: Myocardial Infarction Registry
SMS: single‐marker strategy

**Clinical Perspective**

**What Is New?**

- This study demonstrates that dual‐marker strategies combining high‐sensitivity cardiac troponin T with cardiac myosin‐binding protein C or copeptin are highly effective alternatives to single‐marker protocols and the European Society of Cardiology 0/1‐hour and 0/3‐hour algorithms for non–ST‐segment–elevation myocardial infarction rule‐out.

**What Are the Clinical Implications?**

- Dual‐marker strategies can improve diagnostic efficiency, reduce missed non–ST‐segment–elevation myocardial infarction diagnoses, and optimize emergency department workflows by minimizing the need for sequential blood draws.

The rapid and accurate diagnosis of acute myocardial infarction (AMI) is crucial for effective patient management in an emergency department (ED).^1^ High‐sensitivity cardiac troponin T (hs‐cTnT) and high‐sensitivity cardiac troponin I have improved the diagnosis and management of suspected AMI.^2^, ^3^ In particular, high‐sensitivity cardiac troponin (hs‐cTn) assays have enabled faster diagnosis and management of non–ST‐segment–elevation acute coronary syndrome. Current 2023 European Society of Cardiology (ESC) guidelines recommend the preferential use of the ESC 0/1‐hour or ESC 0/2‐hour algorithm over serial measurements based on the 99th percentile upper limit of normal (ULN) and do not recommend the use of other biomarkers such as copeptin or myosin‐binding protein C (cMyBP‐C), unless hs‐cTn or fast protocols are not available.^4^ ESC protocols allow an immediate rule‐out of non–ST‐segment–elevation myocardial infarction (NSTEMI) in the presence of a low hs‐cTn less than the limit of detection (LoD) (ESC 0‐hour).^4^ There is substantial evidence supporting the clinical performance and safety of strategies that accurately rule out NSTEMI if hs‐cTn is below the limit of blank (LoB) or LoD,^5^, ^6^, ^7^ provided that early presenters have been excluded.

In crowded EDs, the serial measurement of hs‐cTn at prespecified exact time intervals is hampered by time delays to blood draws due to workload or staff exhaustion explaining in part why ESC 0/1‐hour algorithm is associated with poor protocol adherence, protocol violations, and misclassification.^8^, ^9^, ^10^, ^11^, ^12^ Dual‐marker strategies (DMSs) that combine the attractiveness of a single blood draw at presentation with the diagnostic performance of a serial troponin protocol are of potential interest. While most studies have compared DMSs against serial cardiac troponin at the 99th percentile ULN, recent evidence suggests similar diagnostic and prognostic performance, but 2.5‐fold higher effectiveness compared with single marker strategies (SMSs) at the LoD and noninferior performance of DMS compared with ESC 0/1‐hour algorithm.^7^, ^13^, ^14^, ^15^ Recently, cMyPB‐C, a highly abundant cardiospecific molecule, was reported to provide comparable sensitivity and negative predictive values (NPVs) for rapid AMI rule‐out compared with hs‐cTnT/high‐sensitivity cardiac troponin I, with superior performance in triaging patients, especially those presenting within 3 hours of chest pain onset.^16^, ^17^ Findings on the usefulness of DMSs combining cMyPB‐C with copeptin, or cMyPB‐C with hs‐cTn are sparse. Therefore, we sought to compare SMSs and DMSs that use cMyPB‐C, copeptin, or both against a serial hs‐cTnT protocol using the ESC 0/1‐hour and ESC 0/3‐hour algorithm.

## Methods

The data that support the findings of this study are available from the corresponding author upon reasonable request.

### Study Population and Design

The present study uses data from the single‐center ongoing prospective all‐comer MIR (Myocardial Infarction Registry) study.^7^, ^18^ The study population consisted of patients aged ≥18 years presenting with acute symptoms suggestive of acute coronary syndrome (ACS) to the ED of Heidelberg University Hospital from August 2014 to February 2023. Diagnosis included NSTEMI and unstable angina pectoris as well as noncoronary cardiac and noncardiac conditions (non‐ACS). Three different single‐rule‐out protocols based on cMyPB‐C and copeptin were compared against single hs‐cTnT less than LoB or hs‐cTnT less than LoD. In addition, 11 different combinations of DMSs combining copeptin with hs‐cTnT, copeptin with cMyPB‐C, or hs‐cTnT with cMyPB‐C were tested against the ESC 0‐hour, ESC 0/1‐hour, and ESC 0/3‐hour algorithms.

The primary end point was diagnosis of NSTEMI. Additional end points included effectiveness of the respective protocol and safety, defined as numbers of missed NSTEMI (rate of false negatives) at index presentation or combined end point of death, myocardial infarction MI, and stroke at 30 days and 1 year. The comparison of biomarker algorithms for rapid rule‐out of NSTEMI included cMyBP‐C (<10 ng/L, <2.3 ng/L), copeptin (<10 pmol/L), the hs‐cTnT LoB (<3 ng/L) or LoD (<5 ng/L), the hs‐cTnT 99th percentile (≤14 ng/L), and DMSs combining copeptin (<10 pmol/L) with hs‐cTnT (≤14 ng/L, LoB, LoD), cMyBP‐C (<10 ng/L, <2.3 ng/L) with hs‐cTnT (≤14 ng/L, LoB, LoD), and copeptin (<10 pmol/L) with cMyBP‐C (<2.3 ng/L, <10 ng/L). Additionally, the ESC 0/1‐hour algorithm and the ESC 0/3‐hour algorithm were evaluated.^4^, ^19^

NSTEMI without further differentiation into subtypes was diagnosed in clinical routine using the fourth universal definition of MI,^2^ and all diagnoses were retrospectively readjudicated by 3 cardiologists (M.Y., M.M.H., and E.G.) for research purposes. Diagnostic and therapeutic decisions were made at the discretion of the attending physician.

The study was carried out according to the principles of the Declaration of Helsinki and approved by the local ethics committee. Written informed consent was obtained from all patients. The trial was registered on ClinicalTrials.gov (Identifier: NCT06128317).

### Laboratory Analysis

Blood samples for biomarker measurement were collected upon ED arrival and at 1 hour and 3 hours thereafter. Plasma samples were obtained from all patients at baseline for hs‐cTnT, cMyBP‐C, and copeptin. Serial sampling ceased once a diagnosis of AMI was confirmed and before patients underwent coronary angiography or revascularization procedures. Following centrifugation, samples were frozen at −80 °C until further measurement in a blinded fashion.

Measurement of hs‐cTnT in plasma samples was performed using the Elecsys Troponin T high‐sensitive assay (Roche Diagnostics) on a Cobas e411 immunoassay analyzer. LoB, LoD, 10% coefficient of variation and 99th percentile cutoff values were determined to be 3 ng/L, 5 ng/L, 13 ng/L, and 14 ng/L.^20^, ^21^ N‐terminal pro‐B‐type natriuretic peptide (Siemens Atellica IM NT‐proBNP) was measured along with routine blood chemistry from fresh plasma.

cMyBP‐C was assayed under blind conditions in a laboratory at Roche Diagnostics, Penzberg, Germany. The cutoff value for cMyBP‐C was set at ≤2.3 ng/L on the basis of the optimal receiver operating characteristic–derived cutoff value within our cohort (sensitivity ≥99.5% and specificity >95%), and at <10 ng/L as previously described.^22^

Copeptin in plasma samples at baseline (0 hour) was measured with the copeptin pro‐ arginine vasopressin assay on the KRYPTOR compact plus (BRAHMS Thermo Fisher Scientific). Detection limit, precision at 20% coefficient of variation, and 95th percentile cutoff values for the copeptin pro‐arginine vasopressin assay were 0.69 pmol/L, 1.08 pmol/L, and 9.8 pmol/L, respectively.^23^, ^24^

### Statistical Analysis

Continuous variables are presented as median with interquartile range for a nonnormal distribution or as means±95% CIs for normally distributed data. For comparison of continuous parameters, the Mann–Whitney *U* test was used, whereas a χ^2^ test was applied for categorical parameters. Diagnostic performance was assessed in terms of sensitivities, specificities, positive predictive values, NPVs, and receiver operating characteristic curves, comparing areas under the curve using the DeLong test.^25^ All hypothesis testing was 2‐tailed, and *P* values <0.05 were considered statistically significant. All statistical analyses were carried out using R software version 4.3.0 (R Foundation for Statistical Computing, Vienna, Austria) and MedCalc 20.111 (MedCalc Software bvba, Ostend, Belgium).

## Results

### Baseline Characteristics

**Figure 1:**
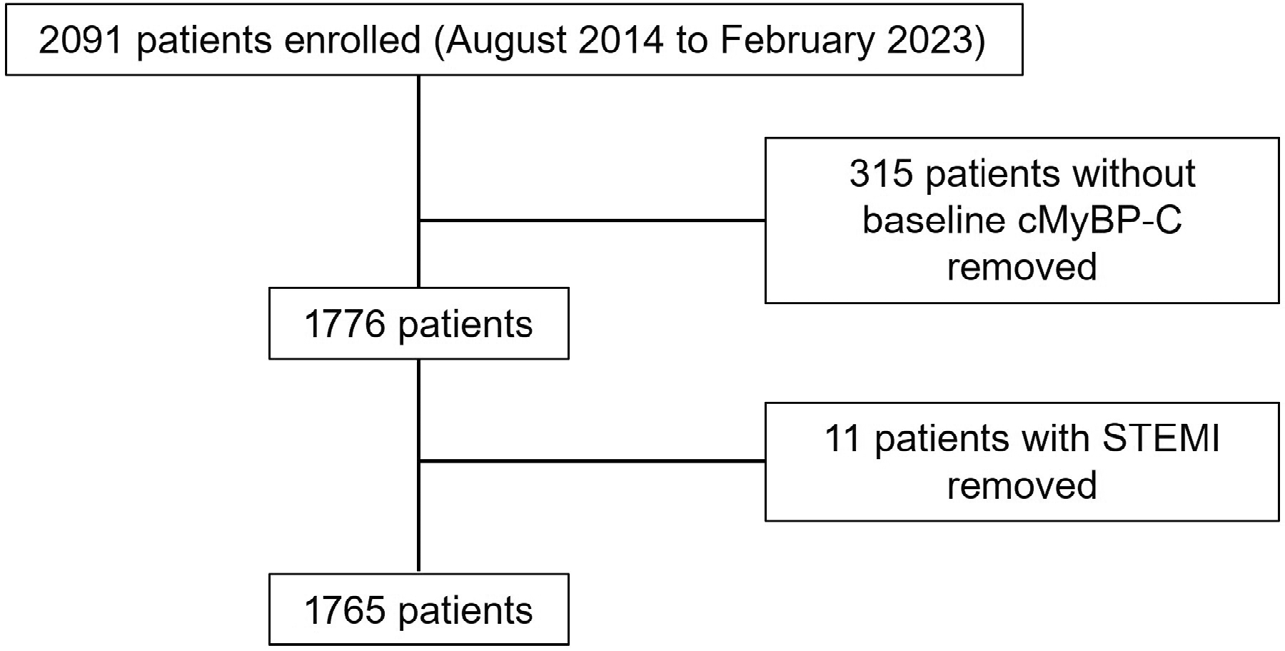
Flowchart of included and excluded patients. This flowchart outlines the recruitment, inclusion, and exclusion processes for patients from 2014 to 2023. cMyBP‐C indicates cardiac myosin‐binding protein C; and STEMI, ST‐segment–elevation myocardial infarction.

The MIR study initially included 2091 patients with suspected ACS, of whom 1765 were eligible for analysis after excluding those with missing baseline cMyBP‐C values and patients with ST‐segment–elevation MI. A consort diagram illustrating the composition of the final study cohort is provided in Figure 1. Baseline characteristics are listed in Table 1. Of the 1765 patients presenting with suspected ACS, 212 (12%) were diagnosed with NSTEMI, 515 (29.2%) with unstable angina pectoris, and 1038 (58.8%) with non‐ACS conditions. Among the entire cohort, 1140 (64.6%) patients had hs‐cTnT levels below the 99th percentile, while 705 (39.9%) had cMyBP‐C levels <10 ng/L, and 1239 (70.2%) copeptin <10 pmoL/L. Most patients presented with chest pain (81.3%), while dyspnea was reported in 42.3% of cases. The median sampling interval between the admission (0 hour) and 1‐hour blood draw was 64 minutes (interquartile range, 60–71), compared with the targeted 60‐minute time frame. The median GRACE (Global Registry of Acute Coronary Events) score was 95.9 (interquartile range, 73.8–119), with 89.2% of patients having a GRACE score <140. Coronary angiography was performed in 34.3% of the entire cohort, among these a significant coronary artery stenosis of ≥70% luminal obstruction was found in 70.2%. Rates of revascularization were 52.6% (percutaneous coronary intervention in 45.4% and coronary artery bypass graft in 8.9%).

**Table 1:** Baseline Characteristics of All Patients

	All patients (N=1765)
Age, y, median (IQR)	66 (55–76)
Female sex, n (%)	671 (38)
Heart rate, bpm, median (IQR)	77 (66–89)
Systolic pressure, mm Hg, median (IQR)	150 (138–165)
GRACE score, median (IQR)	95.9 (73.8–119)
GRACE score <140, n (%)	1574 (89.2)
Diagnosis	
ACS, n (%)	727 (41.1)
NSTEMI, n (%)	212 (12)
Unstable angina pectoris, n (%)	515 (29.2)
Cardiac noncoronary, n (%)	425 (24.1)
Noncardiac, n (%)	613 (34.7)
Symptoms	
Time since onset <3 h, n (%)	236 (13.4)
Chest pain, n (%)	1435 (81.3)
Dyspnea, n (%)	746 (42.3)
Laboratory	
cMyBP‐C, ng/L, median (IQR)	13.7 (5.7–43.5)
Copeptin, pmol/L, median (IQR)	6.0 (3.6–11.7)
hs‐cTnT 0 h, ng/L, median (IQR)	10 (6–20)
Creatinine, mg/dL, median (IQR)	0.9 (0.7–1.0)
NT‐proBNP, ng/L, median (IQR)	233 (74–1228.5)
History	
Myocardial infarction, n (%)	341 (19.3)
Congestive heart failure, n (%)	135 (7.7)
Smoking current, n (%)	314 (18)
Smoking past, n (%)	637 (36.5)
Hypertension, n (%)	1279 (72.5)
Diabetes, n (%)	299 (17)
Dyslipidemia, n (%)	941 (53.3)
Renal disease, n (%)	226 (12.8)
COPD, n (%)	107 (6.1)
Atrial fibrillation, n (%_all_)	356 (20.2)
Diagnostic workup	
Coronary angiography, n (%)	605 (34.3)
Coronary stenosis ≥70%, n (%)	424 (70.2)
Revascularization, n (%)	318 (52.6)
PCI/stent, n (%)	264 (45.4)
CABG, n (%)	54 (8.9)

Percentages may not total 100 because of rounding. ACS indicates acute coronary syndrome; AMI, acute myocardial infarction; CABG, coronary artery bypass graft; cMyBP‐C, cardiac myosin‐binding protein C; COPD, chronic obstructive pulmonary disease; GRACE, Global Registry of Acute Coronary Events; hs‐cTnT, high‐sensitivity cardiac troponin T; IQR, interquartile range; NSTEMI, non–ST‐segment–elevation myocardial infarction; NT‐proBNP, N‐terminal pro‐B‐type natriuretic peptide; and PCI, percutaneous coronary intervention.

### Discriminatory Ability of Single Marker Strategies at Baseline (T0) to Diagnose NSTEMI

**Figure 2:**
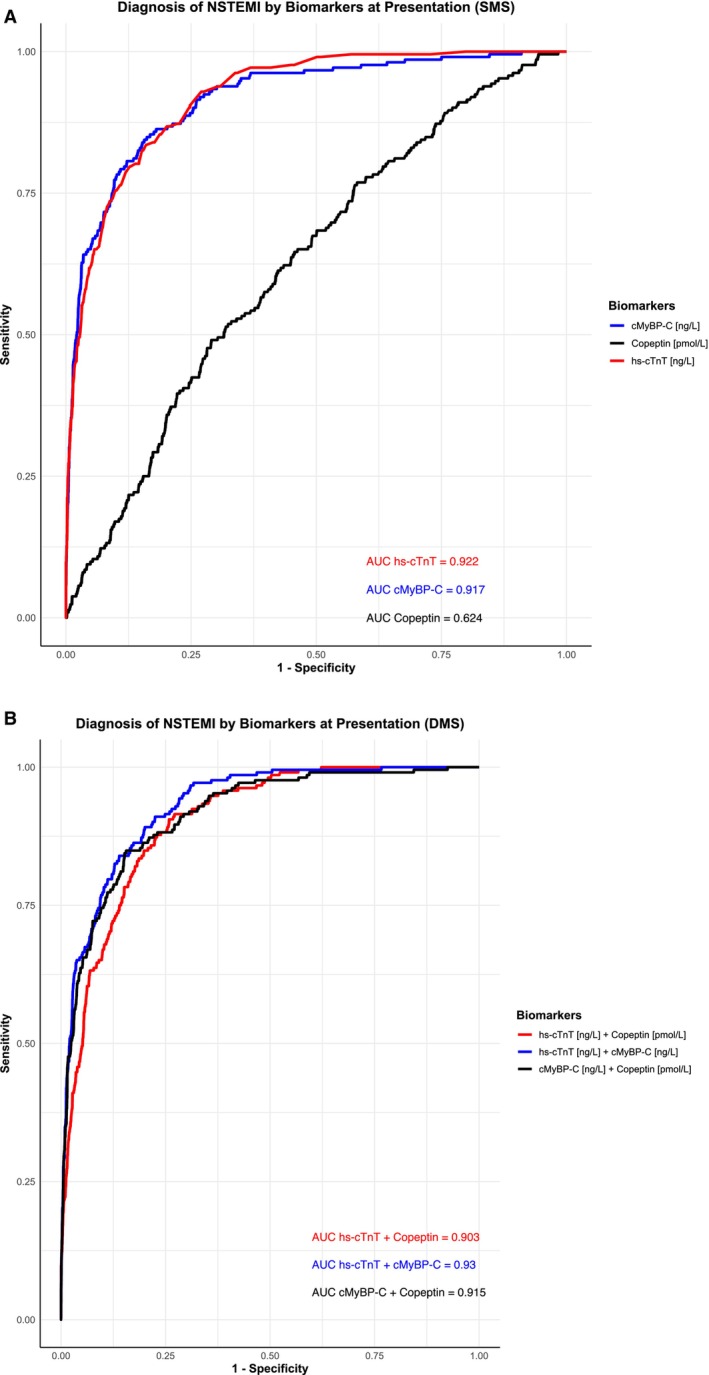
Comparative receiver operating characteristic curves of biomarkers at presentation. **A**, SMS; and (**B**) DMS at 0 hours for diagnosis of NSTEMI. cMyBP‐C indicates cardiac myosin‐binding protein C; DMS, dual‐marker strategy; hs‐cTnT, high‐sensitivity cardiac troponin T; NSTEMI, non–ST‐segment–elevation myocardial infarction; and SMS, single‐marker strategy.

**Figure 3:**
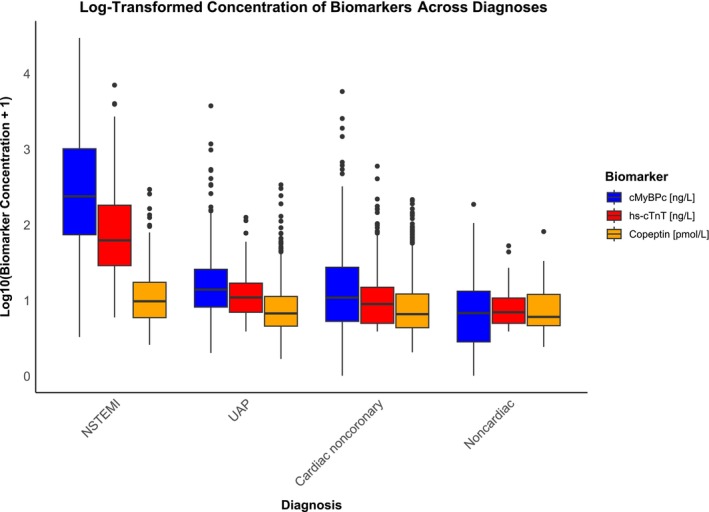
Log‐transformed baseline concentration of cMyBP‐C, hs‐cTnT, and copeptin across diagnoses. cMyBP‐C indicates cardiac myosin‐binding protein C; hs‐cTnT, high‐sensitivity cardiac troponin T; NSTEMI, non–ST‐segment–elevation myocardial infarction; and UAP, unstable angina pectoris.

The area under the curve of hs‐cTnT at presentation for an NSTEMI diagnosis was 0.922 (95% CI, 0.905–0.939), which was comparable with cMyBP‐C, with an area under the curve of 0.917 (95% CI, 0.896–0.938). Not unexpectedly, copeptin in isolation had significantly lower discrimination, with an area under the curve of 0.624 (95% CI, 0.586–0.663). Comparative receiver operating characteristic curves for each biomarker for an NSTEMI diagnosis are shown in both SMSs (Figure 2A) and DMSs (Figure 2B). Additionally, Figure 3 displays boxplots of log‐transformed baseline hs‐cTnT, cMyBP‐C, and copeptin values across different diagnostic groups, highlighting higher baseline concentrations of cMyBP‐C compared with hs‐cTnT in the NSTEMI group.

Table 2 displays the diagnostic performance of each rule‐out strategy. At cutoff values of <3 ng/L (LoB) and <5 ng/L (LoD), hs‐cTnT was associated with sensitivities and NPVs of 100%, low false‐negative rate (FNR) of 0% for both, but a low effectiveness (eligibility rate) of 6.9% and 17.7%, respectively. Hs‐cTnT at the 99th percentile ULN (≤14 ng/L) demonstrated a sensitivity of 92.9%, an NPV of 98.7%, an an effectiveness of 64.6%, but a higher FNR of 7.1%.

**Table 2:** Diagnostic Performance of Each Rule‐Out Strategy

	NPV, % (95% CI)	Sensitivity, % (95% CI)	PPV, % (95% CI)	Specificity, % (95% CI)	Effectiveness, %	FPR, % (95% CI)	FNR, % (95% CI)
Serial measurement strategies							
ESC 0/1‐h algorithm	100 (99.0–100)	100 (97.7–100)	30.3 (28.6–32.0)	63.5 (60.5–66.5)	54.8	36.5 (32.9–40.4)	0.0 (0–2.3)
ESC 0/3‐h algorithm	99.3 (98.5–99.7)	96.8 (93.1–98.8)	40.5 (37.9–43.1)	76.4 (73.8–78.8)	66	23.6 (20.9–26.6)	3.2 (1.2–7.0)
Single measurement strategy at 0 h							
hs‐cTnT <3 ng/L, LoB	100.0 (99.0–100)	100.0 (98.3–100)	12.9 (12.7–13.1)	7.8 (6.5–9.2)	6.9	92.2 (87.5–97.1)	0.0 (0–1.7)
hs‐cTnT <5 ng/L, LoD	100.0 (99.0–100)	100.0 (98.3–100)	14.6 (14.3–14.9)	20.1 (18.1–22.2)	17.7	79.9 (75.5–84.5)	0.0 (0–1.7)
hs‐cTnT ≤14 ng/L, 99th percentile	98.7 (97.9–99.2)	92.9 (88.6–96.0)	31.5 (29.6–33.5)	72.4 (70.2–74.7)	64.6	27.6 (25–30)	7.1 (3.4–11.7)
Copeptin <10 pmol/L	90.7 (89.6–91.7)	45.8 (38.9–52.7)	18.4 (16.1–21.1)	72.4 (70.1–74.6)	70.2	27.6 (25.1–30.4)	54.3 (44.8–65.1)
MyBPC3 <10 ng/L	99.2 (98.1–99.6)	97.2 (93.9–99.0)	19.4 (18.7–20.4)	45.0 (42.5–47.5)	39.9	55.0 (51.4–58.8)	2.8 (1.0–6.2)
MyBPC3 <2.3 ng/L	99.4 (98.1–99.8)	98.6 (95.9–99.7)	16.2 (15.7–16.7)	30.4 (28.1–32.8)	26.9	69.6 (65.5–73.9)	1.4 (0.3–4.1)
DMS Copeptin <10 pmol/L+ hs‐cTnT ≤14 ng/L	99.1 (98.3–99.6)	96.2 (92.7–98.4)	24.2 (23.1–25.5)	.0 (56.4–61.4)	52.3	41.1 (38.0–44.4)	3.8 (1.6–7.4)
Copeptin <10 pmol/L+ LoB	100 (99.0–100)	100 (98.3–100)	12.8 (12.7–13.0)	7.1 (5.9–8.5)	6.2	92.9 (88.2–97.8)	0.0 (0–1.7)
Copeptin <10 pmol/L+ LoD	100 (99.0–100)	100 (98.3–100)	14.2 (14.0–14.5)	17.8 (15.9–19.7)	15.7	82.2 (77.8–86.9)	0.0 (0–1.7)
MyBPC3 <2.3 ng/L+hs‐cTnT ≤14 ng/L	100 (99.0–100)	100 (98.3–100)	16.3 (15.9–16.7)	29.8 (27.6–32.2)	26.2	70.2 (66.1–74.5)	0.0 (0–1.7)
MyBPC3 <10 ng/L+hs‐cTnT ≤14 ng/L	99.9 (99.0–100)	99.5 (97.4–100)	19.4 (18.7–20.1)	43.7 (41.2–46.2)	38.5	56.3 (52.7–60.2)	0.9 (0.1–3.4)
MyBPC3 <2.3 ng/L+LoB	100 (99.0–100)	100 (98.3–100)	12.7 (12.6–12.9)	6.4 (5.3–7.8)	5.7	93.6 (88.8–98.5)	0.0 (0–1.7)
MyBPC3 <10 ng/L+LoB	100 (99.0–100)	100 (98.3–100)	12.9 (12.7–13.0)	7.7 (6.4–9.1)	6.7	92.3 (87.6–97.2)	0.0 (0–1.7)
MyBPC3 <2.3 ng/L+LoD	100 (99.0–100)	100 (98.3–100)	13.9 (13.6–14.1)	15.1 (13.4–17.0)	13.3	84.9 (80.4–89.6)	0.0 (0–1.7)
MyBPC3 <10 ng/L+LoD	100 (99.0–100)	100 (98.3–100)	14.4 (14.1–14.7)	18.9 (17.0–20.9)	16.6	81.1 (76.7–85.7)	0.0 (0–1.7)
Copeptin <10 pmol/L+MyBPC3 <2.3 ng/L	99.5 (98.1–99.9)	99.1 (96.6–99.9)	15.6 (15.2–16.1)	26.9 (24.7–29.2)	23.8	73.1 (68.9–77.5)	0.9 (0.1–3.4)
Copeptin <10 pmol/L+ MyBPC3 <10 ng/L	99.7 (98.7–99.9)	99.1 (96.6–99.9)	18.2 (17.6–18.8)	39.1 (36.7–41.6)	34.5	60.9 (57.1–64.9)	0.9 (0.1–3.4)

CV indicates coefficient of variation; DMS, dual marker strategy; ESC, European Society of Cardiology; FNR, false negative rate; FPR, false positive rate; hs‐cTnT, high‐sensitivity cardiac troponin T; LoB, limit of blank; LoD, limit of detection; MyBPC3, myosin‐binding protein C; NPV, negative predictive value; and PPV, positive predictive value.

Compared with hs‐cTnT ≤14 ng/L, copeptin exhibited lower sensitivity (45.8% versus 92.9%) and lower NPV (90.7% versus 98.7%), and a higher FNR (54.3% versus 7.1%), but had a higher effectiveness (70.2% versus 64.6%). A cMyBP‐C cutoff <10 ng/L was associated with a sensitivity of 97.2%, an NPV of 99.2%, an FNR of 2.8%, and an effectiveness of 39.9%. At a cMyBP‐C cutoff of <2.3 ng/L, sensitivity was 98.6%, NPV was 99.4%, and FNR was 1.4%, but effectiveness declined to 26.9%. Compared with hs‐cTnT at LoB and LoD, sensitivities were slightly lower (97.2% for cMyBP‐C <10 ng/L and 98.6% for cMyBP‐C <2.3 ng/L versus 100% for hs‐cTnT at LoB and LoD) as were NPVs (99.2% for cMyBP‐C <10 ng/L, 99.4% for cMyBP‐C for <2.3 ng/L versus 100% for hs‐cTnT at LoB and LoD). Rates of FNR were higher (2.8% for cMyBP‐C <10 ng/L and 1.4% for cMyBP‐C <2.3 ng/L versus 0% for hs‐cTnT at LoB and LoD) but cMyPB‐C offered higher effectiveness (39.9% for cMyBP‐C <10 ng/L and 26.9% for cMyBP‐C <2.3 ng/L versus 6.9% for hs‐cTnT at LoB and 17.7 for hs‐cTnT at LoD).

### Diagnostic Performance of DMSs Versus Serial hs‐cTnT as the Reference

The serial ESC 0/3‐hour hs‐cTnT strategy yielded a sensitivity of 96.8%, an NPV of 99.3%, an FNR of 3.2%, and an effectiveness of 66.0%.

The serial ESC 0/1‐hour hs‐cTnT strategy showed 100% sensitivity, 100% NPV, a 0% FNR, and an effectiveness of 54.8%.

A total of 11 different DMSs were compared against the serial ESC 0/3‐hour and 0/1‐hour algorithm (Table 2):

1. Compared with the serial ESC 0/3‐hour strategy, a DMS combining hs‐cTnT at the 99th percentile ULN, LoB, and LoD with copeptin (<10 pmol/L) showed comparable sensitivities (96.2%–100% versus 96.8%), NPVs (99.1%–100% versus 99.3%), and FNRs (0%–3.8% versus 3.2%) but lower effectiveness (6.2%–52.3% versus 66%).
2. Compared with the serial ESC 0/1‐hour strategy, a DMS combining hs‐cTnT at the 99th percentile ULN, LoB, and LoD with copeptin (<10 pmol/L) showed variable performance depending on the combination. When combined with LoB and LoD, performance was comparable with the ESC 0/1‐hour strategy but with lower effectiveness (6.2%–15.7% versus 54.8%), while combination with hs‐cTnT at the 99th percentile showed lower overall performance but comparable effectiveness (52.3% versus 54.8%).
3. Compared with the serial ESC 0/3‐hour algorithm, a DMS combining hs‐cTnT at the 99th percentile ULN with cMyBP‐C <10 ng/L yielded improved sensitivity (99.5% versus 96.8%), NPV (99.9% versus 99.3%), and FNRs (0.9% versus 3.2%) but lower effectiveness (38.5% versus 66%).
4. Compared with the serial ESC 0/3‐hour algorithm, a DMS combining hs‐cTnT at the 99th percentile ULN with cMyBP‐C <2.3 ng/L showed improved performance, with sensitivity and NPV at 100 versus 96.8% and 99.3%, respectively. FNR was lower (0.0% versus 3.2%) but effectiveness was reduced (26.2% versus 66%).
5. Compared with the serial ESC 0/1‐hour algorithm, a DMS combining hs‐cTnT at the 99th percentile ULN with cMyBP‐C <10 ng/L demonstrated comparable sensitivity (99.5% versus 100%), NPVs (99.9% versus 100%), FNR (0.9% versus 0%) but lower effectiveness (38.5% versus 54.8%).
6. Compared with the serial 0/1‐hour algorithm, a DMS combining hs‐cTnT at the 99th percentile ULN with cMyBP‐C <2.3 ng/L yielded identical sensitivity, NPV, and FNR (100% and 0%, respectively) but lower effectiveness (26.2% versus 54.8%).
7. Compared with the serial ESC 0/3‐hour algorithm, a DMS combining copeptin <10 pmol/L with cMyBP‐C <10 ng/L improved sensitivity (99.1% versus 96.8%), NPV (99.7% versus 99.3%), and FNR (0.9% versus 3.2%) but had lower effectiveness (34.5% versus 66%).
8. Compared with the serial 0/3‐hour algorithm, a DMS combining copeptin <10 pmol/L with cMyBP‐C <2.3 ng/L yielded comparable overall performance, with sensitivity (99.1% versus 96.8%), NPV (99.5 versus 99.3%), and FNRs (0.9% versus 3.2%) but lower effectiveness (23.8% versus 66%).
9. Compared with the serial 0/1‐hour algorithm, a DMS combining copeptin <10 pmol/L with cMyBP‐C <10 ng/L showed comparable sensitivity (99.1% versus 100%), NPV (99.5 versus 100%), and FNR (0.9% versus 0%) but lower effectiveness (34.5% versus 54.8%).
10. Compared with the serial 0/1‐hour algorithm, a DMS combining copeptin <10 pmol/L with cMyBP‐C <2.3 ng/L yielded comparable sensitivity (99.1% versus 100%), NPV (99.5 versus 100%), and FNR (0.9% versus 0%) but lower effectiveness (23.8% versus 54.8%).

Overall, the serial ESC 0/1‐hour hs‐cTnT strategy demonstrated the highest sensitivity (100%) and NPV (100%), while several DMS incorporating copeptin and cMyBP‐C improved sensitivity and NPV compared with the ESC 0/3‐hour algorithm. However, all DMS strategies had lower effectiveness than both the ESC 0/3‐hour and ESC 0/1‐hour serial algorithms, despite showing comparable or superior diagnostic performance in terms of sensitivity, NPV, and FNR. A DMS combining hs‐cTnT at the 99th percentile ULN with cMyBP‐C <2.3 ng/L or cMyBP‐C <10 ng/L, as well as a DMS combining copeptin <10 pmol/L with cMyBP‐C <10 ng/L, demonstrated superior diagnostic performance compared with the ESC 0/3‐hour algorithm and similar or comparable performance with the ESC 0/1‐hour algorithm, particularly in terms of sensitivity and NPV.

### Event Rates at 30 Days Associated With Single Rule‐Out and DMS Protocols

**Figure 4:**
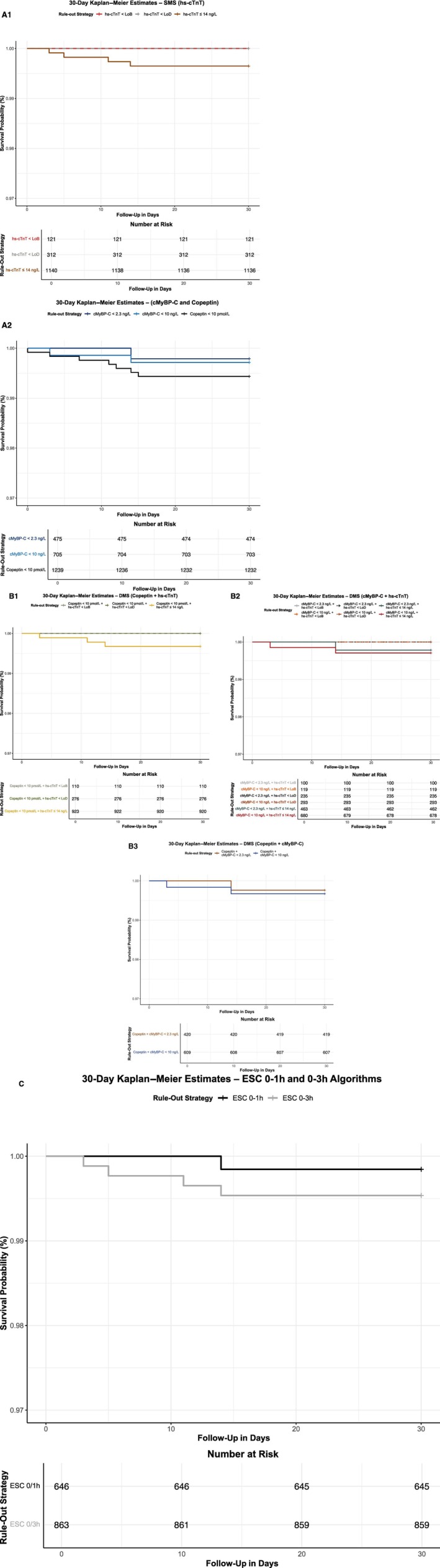
Thirty‐day Kaplan–Meier survival estimates stratified by rule‐out strategy. **A1–A2**, SMS; and (**B1–B3**) DMS compared with ESC 0/1‐hour and ESC 0/3‐hour algorithms (**C**). Kaplan–Meier curves show the cumulative incidence of the combined end point of death, myocardial infarction, or stroke within 30 days in patients ruled out at index presentation. **A1** shows hs‐cTnT–based strategies using the 99th percentile (≤14 ng/L), LoD (<5 ng/L), and LoB (<3 ng/L). **A2** shows strategies based on cMyBP‐C (<10 ng/L and <2.3 ng/L) and copeptin (<10 pmol/L). **B1** shows copeptin (<10 pmol/L) combined with hs‐cTnT (≤14 ng/L, LoD, or LoB). **B2** shows cMyBP‐C (<10 ng/L or <2.3 ng/L) combined with hs‐cTnT (≤14 ng/L, LoD, or LoB). **B3** shows copeptin (<10 pmol/L) combined with cMyBP‐C (<10 ng/L or <2.3 ng/L). **C** shows the ESC 0/1 hour and 0/3 hour algorithms for rule‐out based on serial hs‐cTnT measurements. cMyBP‐C indicates cardiac myosin‐binding protein C; DMS, dual‐marker strategy; ESC, European Society of Cardiology; hs‐cTnT, high‐sensitivity cardiac troponin T; LoB, limit of blank; LoD, limit of detection; and SMS, single‐marker strategy.

**Figure 5:**
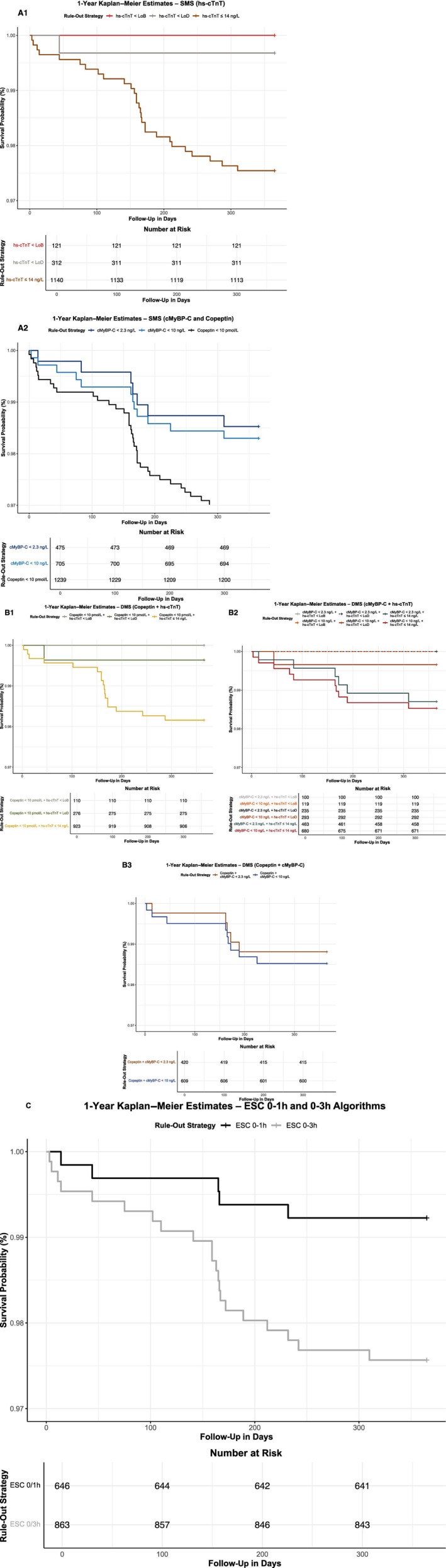
One‐year Kaplan–Meier survival estimates stratified by rule‐out strategy. A1 – A2, SMSs; and (B1 – B3) DMSs compared with ESC 0/1‐hour and ESC 0/3‐hour algorithms (C). Kaplan–Meier curves show the cumulative incidence of the combined end point of death, myocardial infarction, or stroke within 1 year in patients ruled out at index presentation. **A1** shows hs‐cTnT–based strategies using the 99th percentile (≤14 ng/L), LoD (<5 ng/L), and LoB (<3 ng/L). **A2** shows strategies based on cMyBP‐C (<10 ng/L and <2.3 ng/L) and copeptin (<10 pmol/L). **B1** shows copeptin (<10 pmol/L) combined with hs‐cTnT (≤14 ng/L, LoD, or LoB). **B2** shows cMyBP‐C (<10 ng/L or <2.3 ng/L) combined with hs‐cTnT (≤14 ng/L, LoD, or LoB). **B3** shows copeptin (<10 pmol/L) combined with cMyBP‐C (<10 ng/L or <2.3 ng/L). **C** shows the ESC 0/1‐hour and 0/3‐hour algorithms for rule‐out based on serial hs‐cTnT measurements. cMyBP‐C indicates cardiac myosin‐binding protein C; DMS, dual‐marker strategy; ESC, European Society of Cardiology; hs‐cTnT, high‐sensitivity cardiac troponin T; LoB, limit of blank; LoD, limit of detection; and SMS, single‐marker strategy.

Table 3 presents the prognostic performance of each rule‐out strategy, including combined end point of death, MI, and stroke rates at 30 days and 1 year. Overall, 30‐day event rates were low, ranging from 0.0% to 0.6% for SMSs (Figure 4A1 and 4A2) and 0.0% to 0.3% for DMSs (Figure 4B1 through 4B3). In comparison, the 30‐day event rates for the ESC 0/1‐hour and 0/3‐hour algorithms were 0.2% and 0.5%, respectively (Figure 4C). At 1 year, event rates ranged from 0.0% to 3.4% for SMSs and 0.0% to 1.8% for DMSs, compared with 0.8% and 2.4% for the ESC 0/1‐hour and 0/3‐hour algorithms, respectively. Figure 5A1 and 5A2 presents the 1‐year Kaplan–Meier survival curves for SMSs, while Figure 5B1 through 5B3 displays the corresponding curves for DMSs. Figure 5C shows the 1‐year Kaplan–Meier survival curves for the ESC 0/1‐hour and ESC 0/3‐hour algorithms.

**Table 3:** Prognostic Performance of Each Rule‐Out Strategy

	Death/MI/Stroke 30 days		Death/MI/Stroke 1 year	
	% (95% CI)	No.	% (95% CI)	No.
hs‐cTnT <3 ng/L, LoB	0.0 (0–3.1)	0/121	0.0 (0–3.1)	0/121
hs‐cTnT <5 ng/L, LoD	0.0 (0–1.2)	0/312	0.3 (0.01–1.8)	1/312
hs‐cTnT ≤14 ng/L, 99th percentile	0.4 (0.1–0.9)	4/1140	2.5 (1.6–3.6)	28/1140
Copeptin <10 pmol/L	0.6 (0.2–1.2)	7/1239	3.4 (2.4–4.6)	42/1239
MyBPC3 <10 ng/L	0.6 (0.2–1.2)	2/705	1.7 (0.9–3.0)	12/705
MyBPC3 <2.3 ng/L	0.2 (0.0–1.2)	1/475	1.5 (0.6–3.0)	7/475
DMS Copeptin <10 pmol/L+hs‐cTnT ≤14 ng/L (99th perc.)	0.3 (0.1–1.0)	3/923	1.8 (1.1–3.0)	17/923
Copeptin <10 pmol/L+LoB	0.0 (0.0–3.4)	0/110	0.0 (0.0–3.4)	0/110
Copeptin <10 pmol/L+LoD	0.0 (0.0–1.3)	0/276	0.4 (0.01–2.0)	1/276
MyBPC3 <2.3 ng/L+hs‐cTnT ≤14 ng/L	0.2 (0.01–1.2)	1/463	1.3 (0.5–2.8)	6/463
MyBPC3 <10 ng/L+hs‐cTnT ≤14 ng/L	0.3 (0.04–1.1)	2/680	1.5 (0.7–2.7)	10/680
MyBPC3 <2.3 ng/L+LoB	0.0 (0.0–3.7)	0/100	0.0 (0.0–3.7)	0/100
MyBPC3<10 ng/L+LoB	0.0 (0.0–3.1)	0/119	0.0 (0.0–3.1)	0/119
MyBPC3 <2.3 ng/L+LoD	0.0 (0.0–1.6)	0/235	0.0 (0.0–1.6)	0/235
MyBPC3 <10 ng/L+LoD	0.0 (0.0–1.3)	0/293	0.3 (0.01–1.9)	1/293
Copeptin <10 pmol/L+MyBPC3<2.3 ng/L	0.2 (0.0–1.3)	1/420	1.2 (0.4–2.8)	5/420
Copeptin <10 pmol/L+MyBPC3 <10 ng/L	0.3 (0.0–1.2)	2/609	1.5 (1.1–2.8)	9/609
ESC 0/1‐h algorithm	0.2 (0.0–0.9)	1/646	0.8 (0.3–1.8)	5/646
ESC 0/3‐h algorithm	0.5 (0.1–1.2)	4/863	2.4 (1.5–3.7)	21/863

DMS indicates dual marker strategy; ESC, European Society of Cardiology; hs‐cTnT, high‐sensitivity cardiac troponin T; LoB, limit of blank; LoD, limit of detection; MI, myocardial infarction; MyBPC3, myosin‐binding protein C; and NPV, negative predictive value.

## Discussion

In this study, we assessed the diagnostic performance, safety, and effectiveness of different single‐ and dual‐marker algorithms for rule‐out of NSTEMI. In particular, we compared the established ESC 0‐hour SMS at the LoB or LOD versus single rule‐out strategies using c‐MyBP‐C at 2 different low cutoffs, as well as copeptin. In addition, we evaluated the serial ESC 0/3‐hour and ESC 0/1‐hour algorithm against several DMS modifications combining (1) copeptin at a cutoff of 10 pmol/L with hs‐cTnT at the 99th percentile ULN (2) copeptin with cMyPB‐C, and (3) cMyPB‐C combined with hs‐cTnT. In total, 6 SMS and a total of 11 DMSs were tested for diagnostic performance, safety, and effectiveness.

A key feature of our study is that it is the first to directly compare different instant rule‐out strategies including either cMyBP‐C or copeptin or hs‐cTnT alone, as well as DMS combining either biomarker with hs‐cTnT. The ESC 0/1‐hour and ESC 0/3‐hour algorithm served as comparators.

Our evaluation yielded 4 important and novel pieces of information:

Our findings indicate that the discriminatory ability and diagnostic performance of single‐biomarker strategy at presentation to rule out NSTEMI was similar between hs‐cTnT at LoD or LoB and c‐MyBP‐C at either cutoff. Not unexpectedly, effectiveness increased with incremental thresholds. At present, the ESC 0/1‐hour algorithm contains a 0‐hour component that uses hs‐cTn at the LoD or LoB.^4^ Conversely, copeptin as a stand‐alone biomarker showed inferior discriminatory ability for rule‐out of NSTEMI owing mainly to the lack of clinical specificity. Therefore, copeptin has received a recommendation for rule‐out of NSTEMI when used together with cardiac troponin.^26^ Along with the fast ESC algorithms, other validated instant rule‐out algorithms using low hs‐cTn thresholds have been reported.^27^, ^28^, ^29^

Additionally, as the ESC 0/1‐hour or ESC 0/2‐hour algorithm requires repeated troponin measurements at precise time intervals, serial measurements are subject to protocol violations, nonadherence, and potential misclassifications.^8^, ^9^, ^10^, ^11^, ^12^ In addition, the ESC 0/1‐hour and ESC 0/2‐hour protocols use optimized cutoffs and concentration changes that improve rule‐out and rule‐in but still leave a substantial proportion of patients who cannot be triaged and therefore require a third measurement at 3 hours after the initial blood draw.^4^ To reduce time delays associated with inappropriately long turnaround‐time intervals and other obstacles of serial measurements, instant rule‐out strategies using a single measurement of 2 different biomarkers (instead of serial troponin measurements) appear attractive. At the moment, a DMS combining copeptin and cardiac troponin at the 99th percentile ULN has been established as an option if hs‐cTn is not available or in the increasingly uncommon setting that fast protocols cannot be used.^4^ This strategy has been validated in a randomized controlled trial^30^ and prospective observational study^14^, ^23^ to guide the decision of early safe discharge in low‐ to intermediate‐risk patients. DMSs with copeptin have also demonstrated cost effectiveness.^31^ Recently, accumulating evidence suggested that copeptin provides comparable diagnostic performance to the ESC 0/1‐hour algorithm when combined with hs‐cTn.^14^, ^32^, ^33^ The usefulness of cMyPB‐C as part of a DMS when combined with cardiac troponin/hs‐cTn or copeptin has not been explored yet. The findings of our observational study demonstrate similar discriminatory ability and performance of DMSs with the ESC 0/1‐hour algorithm and superior performance compared with the ESC 0/3‐hour algorithm, regardless of whether copeptin is combined with hs‐cTnT or cMyPB‐C. Whether cMyPB‐C can be used as an alternative to hs‐cTnT remains speculative at the moment and requires prospective testing in unselected populations with lower prevalence of non–ST‐segment–elevation ACS in the real world. Currently, it is more attractive to instrument a more effective single‐rule‐out strategy that is based on 2 biomarkers instead of single troponin‐based or serial troponin measurement strategy to overcome logistic shortcoming associated with the appropriate execution of serial sampling in crowded EDs.

Our findings further show that a DMS is able to reduce numbers of missed MIs to similarly low rates as the single hs‐cTn–based 0‐hour algorithm and the ESC 0/1‐hour algorithm, while maintaining higher effectiveness than the 0‐hour algorithm and comparable effectiveness with the serial ESC 0/1‐hour and ESC 0/3‐hour protocols. DMS using hs‐cTnT and c‐MyPB‐c <2.3 ng/L further improves identification of patients at risk. DMSs with hs‐cTnT and c‐MyPB‐c <10 ng/L or the addition of cMyPB‐C to copeptin also demonstrate favorable diagnostic performance in identifying patients at risk. Compared with the ESC 0/3‐hour algorithm, a DMS with copeptin and hs‐cTnT reduces the number of missed MI cases, whereas no improvement was observed compared with the ESC 0/1‐hour algorithm. Additionally, a DMS combining cMyPB‐C <10 ng/L or <2.3 ng/L reduces numbers of missed MIs by 12.5% and 56.25% respectively, while a DMS combining copeptin with cMyPB‐C <10 ng/L or <2.3 ng/L identifies 71.9% of MI cases missed by the ESC 0/3‐hour algorithm.

Finally, our study demonstrates that hs‐cTnT, serial hs‐cTnT, and DMSs provide similar prognostic information, independent of clinical data such as the GRACE score. A clinical survey by Than et al investigated the acceptable risk of major adverse cardiac events in patients with chest pain soon after discharge from the ED and found that the potentially acceptable AMI miss rate should be ≤1%.^34^ Overall, 30‐day event rates were low across all strategies: 0.0% to 0.6% for SMSs, 0.0% to 0.3% for DMSs, 0.2% for the ESC 0/1‐hour algorithm, and 0.5% for the ESC 0/3‐hour algorithm. At 1 year, event rates were also low, ranging from 0.0% to 3.4% for SMSs, 0.0% to 1.8% for DMSs, 0.8% for the ESC 0/1‐hour algorithm, and 2.4% for the ESC 0/3‐hour algorithm. These findings highlight the safety of all rule‐out strategies in short‐term outcomes and their effectiveness in long‐term prognostication from the time of ED presentation.

### Limitations

While our study provides valuable insights into the diagnostic performance of hs‐cTnT, cMyBP‐C, and copeptin, it is essential to acknowledge several limitations.

First, the study was conducted at a single tertiary referral center in Germany, potentially limiting the generalizability of our findings to other geographic regions or health care systems.

Another important consideration is that the diagnostic cutoff for cMyBP‐C (<2.3 ng/L) requires external validation in independent populations. Furthermore, our findings were generated with hs‐cTnT and no blood specimens are available for evaluation of other high‐sensitivity cardiac troponin I assays.

A potential source of bias arises from the use of hs‐cTnT in clinical adjudication, which inherently affects the assessment of cMyBP‐C. Additionally, our study focused on evaluating hs‐cTnT, cMyBP‐C, and copeptin in comparison with the ESC 0‐hour and the serial ESC 0/1‐hour algorithm and ESC 0/3‐hour algorithm. No other protocols such as accelerated diagnostic protocols ESC 0/2‐hour algorithm or HighSTEACS (High‐Sensitivity Troponin in the Evaluation of Patients With Suspected Acute Coronary Syndrome) algorithm^35^ or other potential biomarkers or risk scores were investigated.

Moreover, it should be noted that cMyBP‐C is not yet commercially available as a routine assay, which limits its immediate clinical applicability.

Finally, given that fast protocols accelerate diagnosis and management in low‐risk patients and due to the availability of blood specimen for the initial blood draw, we were unable to test the performance of serial copeptin or serial c‐MyBP‐C. In addition, we focused on rule‐out due to restrictions regarding the intended use of a DMS with copeptin in combination with hs‐cTn.

### Conclusions

Our study provides valuable insights into the diagnostic performance of DMSs for NSTEMI rule‐out, demonstrating safety and effectiveness comparable with established ESC algorithms. Specifically, DMS strategies combining hs‐cTnT with either cMyBP‐C or copeptin, offer promising alternatives to hs‐cTnT‐only protocols by reducing missed MI rates and enhancing identification of high‐risk patients, without compromising short‐ or long‐term prognostication. These strategies could streamline clinical workflows by minimizing the need for sequential sampling required in the ESC 0/1‐hour and 0/3‐hour algorithms, while maintaining similar diagnostic accuracy. However, further validation across diverse patient populations and clinical settings is necessary to confirm these findings and ensure long‐term safety and effectiveness.

## Sources of Funding

The study was supported by a research grant from Roche Diagnostics. The sponsor had no influence on the study concept, data collection, or interpretation. For the publication fee, we acknowledge financial support by Heidelberg University.

## Disclosures

M.M.H. received research funding from BRAHMS Thermo Fisher Scientific and Roche Diagnostics and served as a consultant to ZOLL CMS GmbH. E.G. received honoraria for lectures from Roche Diagnostics, AstraZeneca, Bayer, Daiichi‐Sankyo, and Lilly Eli Deutschland. He serves as a consultant for Roche Diagnostics, BRAHMS Thermo Fisher Scientific, and Boehringer Ingelheim and has received research funding from BRAHMS Thermo Fisher Scientific, Roche Diagnostics, Bayer Vital, and Daiichi Sankyo. N.F. has received speaker honoraria from Daiichi Sankyo, Astra Zeneca, Boehringer Ingelheim, and Bayer Vital. There are no disclosures for M.Y. and C.S.
